# Unraveling the Link Between n‐Back Working Memory and Algebraic Ability in Adolescents: Correlations and Training Effects

**DOI:** 10.1002/pchj.70047

**Published:** 2025-08-07

**Authors:** Jingguang Li, Xia Chen, Liyun Hua, Zhidong Wang, Yajun Zhao, Xingbo Wang, Wei Liu

**Affiliations:** ^1^ School of Teacher Education Dali University Dali China; ^2^ Da Yawan No. 1 Middle School Huizhou China; ^3^ School of Education and Psychology Southwest Minzu University Chengdu China; ^4^ School of Education Yunnan Minzu University Kunming China

**Keywords:** adolescents, algebraic ability, n‐back, training, working memory

## Abstract

Individuals with higher working memory capacities excel in mathematics performance. However, limited research has explored the impact of working memory on adolescents' algebraic ability and the transferability of training effects. Therefore, we conducted the current investigation with Chinese adolescents. In a correlational study (*n* = 218), we identified a positive association between n‐back working memory and the ability to solve algebraic word and equation problems. In a subsequent training study, the experimental group (*n* = 28) underwent adaptive n‐back working memory training for 20 days, resulting in enhanced working memory performance. However, no improvements in algebraic performance were observed in the experimental group compared to either the passive control (*n* = 22) or the active control group (*n* = 28). Together, while n‐back working memory performance is associated with better algebraic performance, leveraging training gains of working memory to enhance algebra learning presents challenges. The theoretical and practical implications of these findings are presented.

## 1 Introduction

Algebra, the study of mathematical symbols and the rules for manipulating them, is a fundamental branch of mathematics. Algebraic ability is regarded as the gateway to academic achievement in science, technology, engineering, and mathematics (STEM) fields and is a positive predictor of career success (National Mathematics Advisory Panel 2008). To enhance the teaching and learning of algebra, researchers are interested in understanding the cognitive factors that contribute to individual differences in algebraic ability. By identifying these factors, educators can develop targeted intervention strategies that may have a far‐transfer effect on improving algebraic ability.

The literature has identified several domain‐general and domain‐specific cognitive factors that may influence algebraic ability, including inhibition control (Khng and Lee 2009), knowledge of fractions and division (Siegler et al. 2012), acuity of approximate number representation (Geary et al. 2015), and arithmetic ability (Tolar et al. 2009). Among all the factors that influence algebraic ability, working memory is a particularly promising candidate. Working memory refers to the capacity to temporarily maintain and process information (Baddeley and Hitch 1974). Numerous studies have revealed that working memory performance can positively predict mathematical performance or academic achievement in mathematics (see the meta‐analysis by Peng et al. 2016; see also reviews by Bull and Lee 2014; Cragg and Gilmore 2014; Raghubar et al. 2010). Students who encounter math learning difficulties often demonstrate deficits in working memory (David 2012). Furthermore, meta‐analyses based on dual‐task paradigms have shown that working memory load leads to poorer arithmetic task performance (Chen and Bailey 2021), providing strong evidence of the dependence of mathematical tasks on working memory resources. However, previous studies have not extensively explored the relationship between working memory and algebraic task performance, particularly the potential of working memory training to enhance algebraic ability. Therefore, this study employed a combination of correlational and training methodologies to comprehensively investigate the role of working memory in algebraic ability.

### 1.1 Correlational Studies on the Association Between Working Memory and Algebraic Ability

Compared with numerous studies that have investigated the association between working memory and arithmetic, only a few studies have examined the link between working memory and algebra. Most of these studies have consistently identified a positive correlation between working memory and algebraic proficiency or success, predominantly observed in young children (Cirino et al. 2022; Geary et al. 2015; Lee et al. 2004; Lee et al. 2009; Lee et al. 2011; Lee et al. 2018). Notably, a limited number of studies have reported similar observations among high school (Siegler et al. 2012; Trezise and Reeve 2014) or college students (Tolar et al. 2009). This positive correlation pattern is consistent with a meta‐analysis (Peng et al. 2016) that reported a positive association between working memory performance and algebraic ability (*r* = 0.27, 95% confidence interval (CI) = [0.09, 0.44]). Although evidence of the link between working memory and algebra has been accumulating, several research gaps remain.

First, understanding the link between working memory and algebraic ability requires acknowledgment of the underutilized n‐back paradigm as a measure of working memory in contrast to the complex span tasks more commonly used in prior studies (e.g., Geary et al. 2015; Lee et al. 2004; Lee et al. 2011; Lee et al. 2018; Tolar et al. 2009; Trezise and Reeve 2014). The operational distinctions between the complex span task, which requires recall of information while engaging in a secondary task that serves as a distractor (Daneman and Carpenter 1980), and the n‐back task, which demands the continuous recognition of stimuli following a predefined delay (Cohen et al. 1997), indicate that these tasks might access distinct components of working memory (Redick and Lindsey 2013). Specifically, the two tasks diverge on two key features: (1) complex span tasks demand serial recall, whereas n‐back tasks depend on speeded recognition of the item presented *n* trials earlier (Kane et al. 2007); and (2) complex span performance hinges on maintenance—keeping goal‐relevant items active despite distraction—whereas n‐back success hinges on rapid disengagement, actively dropping outdated items to avoid “far‐lure” false alarms, a mechanism that underpins the task's strong association with updating (Burgoyne et al. 2025). These distinctions are reinforced by the observed weak correlation between performance on the two tasks (Kane et al. 2007; Redick and Lindsey 2013) and the lack of transfer effects as demonstrated in training studies involving both tasks (Chooi and Thompson 2012; Jaeggi et al. 2008; Jaeggi et al. 2010; Kundu et al. 2013; Li et al. 2008; Lilienthal et al. 2013; Richmond et al. 2011). Thus, some scholars urge researchers not to use these two task paradigms interchangeably (Burgoyne et al. 2025; Redick and Lindsey 2013). Additionally, the working memory components measured by the n‐back task—including simultaneous maintenance and manipulation of multiple items and sustained attention—are likewise fundamental to algebraic mental operations. The n‐back task specifically requires updating through rapid disengagement, partly mirroring the overwrite cycle in algebraic tasks (especially during mental calculation), where every operation (e.g., transposing a term or combining like terms) overwrites the previous equation representation and retains only the updated form in working memory. Together, incorporating the n‐back task into the exploration of the relationship between working memory and algebraic ability not only addresses a methodological gap in existing research (i.e., the rare use of the n‐back paradigm) but also aligns with the cognitive processes that are fundamental to algebraic tasks.

Second, although working memory performance is reliably associated with better algebraic outcomes, the cognitive route that connects them remains unclear. We therefore propose a sequential account. Greater working memory performance is expected to enhance arithmetic ability (Peng et al. 2016), because efficient arithmetic computation relies on holding and updating interim quantities. Enhanced arithmetic ability should, in turn, strengthen the ability to solve algebraic equation problems (i.e., solving explicit equations such as 3X + 2 = 11), because isolating an unknown variable requires fluent execution of the basic operations mastered in arithmetic (Tolar et al. 2009). The ability to solve equation problems is then expected to support the ability to solve algebraic word problems (i.e., verbal problems that must be translated into equations before solving), because success on such tasks first involves forming an equation that mirrors the verbal relations and then solving that equation. Testing this proposed sequence will provide a preliminary understanding of the cognitive foundations of adolescent algebraic abilities.

### 1.2 Working Memory Training and Its Effects on Mathematics

Beyond exploring the predictive influence of n‐back working memory on algebraic ability, a pivotal question emerges: Is it possible for the training benefits associated with working memory to be extended to bolster algebraic ability? The presence of such transfer effects would carry profound implications for the educational domain, suggesting that training in working memory might serve as a viable strategy for augmenting algebraic ability.

Many empirical studies have shown that working memory can be trained and that training effects can be transferred to other domains, such as general intelligence (Klingberg et al. 2002), reading (Karbach et al. 2015), decision making (Zhao et al. 2022), and even anhedonia (Zhang et al. 2019). For example, one early seminal study reported that training for the dual n‐back working memory task improved working memory itself and led to an increase in fluid intelligence in healthy young adults (Jaeggi et al. 2008). However, several recent meta‐analyses have cast doubt on the effectiveness of working memory training (Melby‐Lervåg and Hulme 2013; Melby‐Lervåg et al. 2016; Rodas et al. 2024; Sala et al. 2019; Sala and Gobet 2020) by suggesting that it may only have reliable near‐transfer effects (i.e., task‐dependent improvements) but not reliable far‐transfer effects (i.e., improvements in other domains, such as general intelligence).

Moreover, the available evidence—drawn largely from studies involving preschoolers, elementary school pupils, and middle school students (see Lee 2023 for a review)—shows mixed findings regarding the far‐transfer effects of working memory training on foundational mathematical abilities. On the one hand, a considerable body of evidence supports the idea that such training enhances various mathematical performances, including numeracy (Kroesbergen et al. 2014; Muñez et al. 2022), arithmetic performance (Bergman‐Nutley and Klingberg 2014), and overall mathematics achievement (Layes et al. 2018; Söderqvist and Bergman Nutley 2015; Zhang et al. 2018). Conversely, opposing studies present evidence that working memory training does not lead to notable improvements in mathematical performance (Roberts et al. 2016; St Clair‐Thompson et al. 2010). Currently, there is no universally accepted explanation for these inconsistent results. Critics argue that many of the early positive results stemmed from methodological limitations—such as small samples or weak control conditions—whereas more recent, tightly designed experiments rarely detect far‐transfer effects (Lee 2023). An alternative account posits that transfer is most likely when the training task and the mathematical outcome share substantial representational overlap, thereby engaging common cognitive processes (Peng and Swanson 2022). A further complication is that the existing evidence is heavily skewed toward span‐based working memory training, whereas only very limited work has employed an n‐back updating procedure (e.g., Zhang et al. 2018); consequently, it remains unclear whether interventions that target the updating component of working memory would produce similar—or different—transfer effects.

Despite these debates, one clear gap remains: almost no studies have yet directly tested whether working memory training can boost algebraic performance. Addressing this oversight is crucial because, although algebra builds upon arithmetic, the cognitive terrain it occupies is far more demanding. For example, solving an algebraic word problem requires the solver to translate a real‐world narrative into symbolic form, keep track of one or more unknowns, and preserve the balance of an equation through a sequence of operations—processes that impose a markedly heavier load on working memory than the basic arithmetic computation (Ji and Guo 2023). Moreover, algebra is widely recognized as the “gateway course” that largely determines whether students remain on a STEM pathway (National Mathematics Advisory Panel 2008). Consequently, if working memory training can enhance algebraic ability, it would clarify when such training transfers to mathematical fields and, more importantly, offer a tool with high stakes for mathematics education, even if its effects on non‐algebraic mathematical abilities remain contested. Considering the scarce research in this specific area, it is imperative to examine whether working memory training has the potential to significantly enhance algebraic ability.

### 1.3 The Current Investigation

In response to the outlined research gaps, the current investigation aimed to elucidate the relationship between n‐back working memory and algebraic ability in adolescents and to examine the potential benefits of working memory training on algebraic performance in two related studies. Unlike many previous studies, our research focused on adolescents (high school and college students) rather than children (especially elementary school students). This is because adolescents' learning in STEM fields is more heavily reliant on algebraic ability. Additionally, this focus allowed us to design more complex algebraic tests (such as equation problems with multiple unknowns) in our investigation. Consequently, researching adolescents will serve as a crucial supplement to studies involving children.

Specifically, in the correlational investigation (Study 1), we aimed to test the association between n‐back working memory performance and algebraic ability. In addition, we examined the mediating role of arithmetic ability and the ability to solve algebraic equation problems in the link between working memory and the ability to solve algebraic word problems. This examination offers insights into the cognitive underpinnings that may facilitate solving algebraic problems. Based on the findings from Study 1, we assessed the possibility of targeted n‐back working memory training in enhancing algebraic ability in an intervention investigation (Study 2). Participants underwent an adaptive n‐back training procedure. Their performance on algebraic tasks was measured pre‐ and post‐intervention and compared with control groups that received either no training or alternative control interventions.

A key strength of this study is the integration of both correlational and training designs within a single investigation, a practice rarely adopted in the literature in this field. This combination significantly enhances the study's methodological rigor. Establishing a correlation alone does not validate the efficacy of an intervention. Furthermore, the ineffectiveness of an intervention may arise from the selection of a training task that is not suitably predictive of mathematical ability. Overall, our research provides a comprehensive view of how working memory contributes to algebraic ability and rigorously tests the hypothesis that working memory training can enhance mathematical performance. This study may offer novel pedagogical insights to improve algebra education.

## 2 Study 1

The main objective of Study 1 was to assess whether working memory performance, as measured by the n‐back task, can serve as a predictor of adolescents' algebraic ability. If a positive association is identified, we aim to further explore the potential mediating role of arithmetic ability within this relationship.

### 2.1 Methods

#### 2.1.1 Participants and Procedures

The participants for this study were 222 10th grade students recruited from four classes at one local public high school in Dali, China, as part of a larger project with the major goal of investigating the relationship between executive function and academic performance among Chinese adolescents (Li et al. 2020). The mean age of the participants was 16.08 years (SD = 0.54), and 66% of the participants were girls. Using G*Power 3.1 (Faul et al. 2009), we determined that 138 participants would suffice to detect a medium‐sized correlation (*r* = 0.30; Cohen 1988) with α = 0.05 and 95% power. A complementary Monte Carlo simulation (Schoemann et al. 2017) for the planned mediation model indicated that at least 220 participants were required to achieve 75% power at α = 0.05, assuming intercorrelations of *r* = 0.30 (SD = 0.10) among the independent variable, mediators, and outcome variable. Therefore, the sample size in this study was adequate for both correlation and mediation analyses. The Medical Ethics Committee of Dali University approved the study, and we obtained written informed consent from all participants and their parents.

In their classrooms together with their classmates, the participants completed three paper‐based tests: an arithmetic ability test, an algebraic ability test, and Raven's advanced progressive matrices (APM). The number of students in each class ranged from 54 to 55. In addition, the participants individually completed two computer‐based tasks (i.e., the 1‐back and 2‐back tasks) on a different day from the paper‐based tasks.

#### 2.1.2 Tasks

##### 2.1.2.1 N‐Back Task

We used unmodified versions of 1‐back and 2‐back tasks from the well‐validated NIH‐EXAMINER (Executive Abilities: Measures and Instruments for Neurobehavioral Evaluation and Research) test battery to measure working memory (Kramer et al. 2014).

In the n‐back tasks, participants were shown a series of white squares that appeared in 15 different locations on a black screen. Each square was presented for 1000 ms. All of the locations were equidistant from the center of the screen. During the 1‐back task, participants were instructed to judge whether the square was presented in the same or a different location than the previous square by pressing keys. A number (randomly varying from 1 to 9) appeared in the center of the screen 500 ms after each response and remained on screen for 1000 ms. The participants were required to read this number aloud immediately after it appeared before responding to the next square. The number task prevented the participants from visually fixating on the location of the previous square. During the 2‐back task, the participants were instructed to judge whether a presented square appeared in the same or a different location than the square two squares earlier. Unlike the 1‐back task, no numbers were presented between trials in the 2‐back task. The 1‐back task included 30 trials (10 locations were the same and 20 locations were different), and the 2‐back task included 90 trials (30 locations were the same and 60 locations were different).

N‐back tasks are challenging for some individuals. Following the NIH‐EXAMINER instructions, if participants failed to obtain a passing score during the practice session, we did not administer the formal test to them. Due to other commitments or voluntary withdrawal during testing, two students did not participate in the n‐back tasks session. Among the remaining participants (*n* = 220), 216 (98.64%) and 196 (89.09%) of them passed the practice sessions and completed the 1‐back and 2‐back tasks, respectively. The average overall accuracy for the 1‐back and 2‐back tasks was 86.9% and 71.6%, respectively. To comprehensively evaluate the participants' performances, in accordance with the NIH‐EXAMINER (Kramer et al. 2014), we used *d*′ as the measure of working memory in subsequent analyses. Based on signal detection theory, *d*′ was calculated by the participants' ability to discriminate signals from noise. Participants who completed the 2‐back task had higher *d*′ scores on the 1‐back task (*M* = 2.24, SD = 0.64) than those who did not complete the practice session for the 2‐back task (*M* = 1.94, SD = 0.70), *t*(215) = 2.05, *p* = 0.04, Cohen's *d* = 0.47, suggesting performance consistency between the two forms of n‐back tasks. To simplify the subsequent data analyses, the average of the *d*′ scores from the 1‐back and 2‐back tasks was used as the measure of working memory performance. In this study, the split‐half reliability (estimated via the Spearman‐Brown correction) for the overall *d*' was 0.72.

##### 2.1.2.2 Arithmetic Tasks

The calculation fluency test (CFT) was used to index the participants' arithmetic ability (Douglas and LeFevre 2017). The CFT comprises three subtests involving two‐term arithmetic problems on three separate A4 pages. The first test consists of double‐digit addition problems (e.g., 52 + 19), the second test consists of double‐digit subtraction problems (e.g., 84–47), and the third test consists of multiplication problems (e.g., 67 × 4). For each test, participants are given 1 min and instructed to solve the problems quickly and accurately. Each test has six rows of problems with 10 problems per row (i.e., 60 problems for each test). Before the formal test, the participants solved six sample practice problems from each subtest. Given that all the arithmetic questions were easy for all the participants (i.e., 10th grade high school students), the CFT was considered a fluency‐based test. On average, the participants correctly completed 22.0, 17.0, and 16.3 problems on the addition, subtraction, and multiplication subtests, respectively. In this study, the Cronbach's α values for the addition, subtraction, and multiplication subtests were 0.95, 0.97, and 0.97, respectively. Moreover, performances on the three subtests were highly correlated (correlations ranged from 0.68 to 0.72). To simplify the subsequent analyses, the sum of the three subtests performances was used as a measure of arithmetic calculation ability (e.g., Douglas and LeFevre 2017).

##### 2.1.2.3 Algebraic Tasks

Following the CFT test format, we developed two algebraic tasks to assess the participants' algebraic ability. The first task, *algebraic equation solving*, is defined as the procedural ability to solve pure linear algebra equations with one unknown (e.g., “X − 12 = 74; X = ?”). The second task, *algebraic word problem solving*, is defined as the integrated ability to translate a verbally stated quantitative situation into a symbolic linear equation with one unknown and subsequently solve that equation (e.g., “This year, the school planted 50 trees, which is 18 more than the number planted last year. How many trees did the school plant last year?”).

For the algebraic equation solving task, participants solved 60 single unknown linear equations distributed across two A4 sheets. For the algebraic word problem solving task, participants solved 24 word problems—each of which could be represented as a single unknown linear equation—distributed across four A4 sheets. The participants were given 2 min for the algebraic equation solving task and 5 min for the algebraic word problem solving task. For both tasks, the participants were instructed to solve the problems quickly and accurately. Before the formal test, the participants solved three sample practice problems from each subtest. The total number of questions the participants answered correctly on both algebraic tasks was used as a measure of algebraic ability. In this study, the Cronbach's α values for the algebraic equation solving and algebraic word problem tasks were 0.89 and 0.81, respectively.

##### 2.1.2.4 Raven's APM

Raven's APM, in which participants are required to identify a missing figure to complete a larger pattern, is a widely used tool to measure general intelligence. To reduce the participants' burden, an abbreviated form of Raven's APM was used (Bors and Stokes 1998). This test involves only 12 selected items from the original 36‐item Raven's APM but has been shown to retain the difficulties and validity of the original test (Bors and Stokes 1998). Given the limited number of items, the Cronbach's α of the Raven's APM scores for this study was only 0.57. Despite reliability concerns, we included the Raven's APM scores in further analyses for a rough measure of general intelligence.

#### 2.1.3 Data Analysis

All statistical analyses were performed using R 3.4.3 (R Foundation for Statistical Computing, Vienna, Austria). The descriptive, reliability, and correlation analyses were performed with the Psych package (Revelle 2023). The mediation analyses (Baron and Kenny 1986) were conducted using the Lavaan package (Rosseel 2012). To assess the statistical significance of the indirect impact, we employed a bootstrapping technique (Preacher and Hayes 2008) with 5000 samples to produce 95% confidence intervals (CIs). The indirect effect was deemed significant if the CI did not encompass zero. We present standardized regression coefficients in the mediation analyses. Additionally, some participants failed to complete the n‐back working memory test because they did not pass the practice session (*n* = 24), and a small number of participants voluntarily chose to withdraw from the mathematical ability tests (two participants for the CFT and one participant for the algebraic word problem test). The ultimate sample size for the correlation and mediation analyses was 193.

### 2.2 Results and Discussion

**FIGURE 1:**
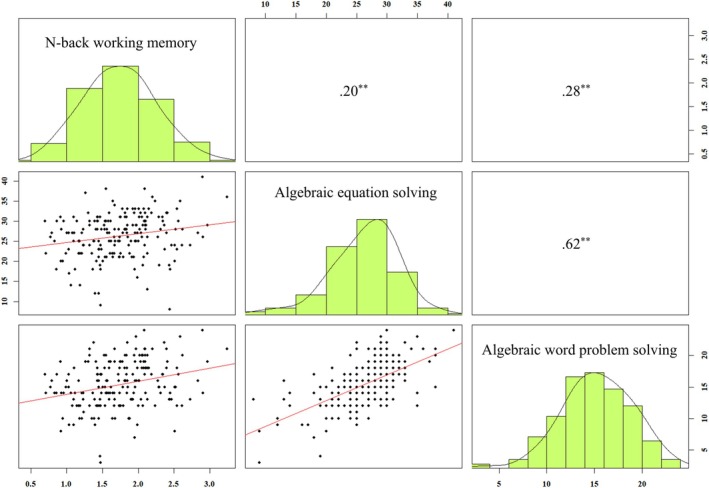
Histograms (located on the diagonal), scatterplots of correlations (below the diagonal), and Pearson correlation coefficients (above the diagonal) for n‐back working memory, algebraic equation solving, and algebraic word problem solving in Study 1. **p < 0.01.

Table 1 presents the means, standard deviations, and Pearson's correlation coefficients for the main study measures. Importantly, n‐back performance was positively correlated with algebraic abilities (for algebraic equation solving, *r* = 0.20, *p* < 0.01; for algebraic word problem solving, *r* = 0.28, *p* < 0.01; Figure 1). After controlling for age and gender, the correlations between n‐back performance and the indicators of algebraic ability remained significant (algebraic equation solving, partial *r* = 0.20, *p* = 0.01; algebraic word problem solving, partial *r* = 0.27, *p* < 0.01). In summary, individuals who performed better on n‐back tasks were also better at solving algebraic problems.

**TABLE 1:** Means, SDs, and correlations among variables in Study 1.

	*M*	SD	1	2	3	4
1. N‐back working memory performance	1.8	0.5	—			
2. Algebraic equation solving ability	26.5	5.3	0.20**	—		
3. Algebraic word problem solving ability	15.5	3.6	0.28**	0.62**	—	
4. Arithmetic ability	55.4	10.5	0.22**	0.46**	0.37**	—
5. General intelligence	6.5	2.1	0.23**	0.13	0.23**	0.05

*Note*: **p < 0.01.

A key follow‐up question concerns the magnitude of the observed association between n‐back performance and algebraic ability. Given that effect sizes in psychological research lack universally accepted benchmarks, we evaluated the strength and significance of this association from three complementary perspectives. First, because task‐based measures inevitably involve measurement error, the observed correlations are likely attenuated. To address this, we corrected for attenuation based on the reliability of the respective measures. Specifically, we estimated the upper bound of the correlation as the square root of the product of the reliability coefficients of the two constructs, and then computed the corrected correlation coefficients by dividing the observed correlations by these upper bounds (Schmidt and Hunter 1999). After correction, the association between n‐back performance and algebraic abilities appeared notably stronger (for algebraic equation solving, *r* = 0.28, accounting for 6.24% of the variance; for algebraic word problem solving, *r* = 0.37, accounting for 13.44% of the variance). Second, the observed effect sizes are consistent with the comprehensive meta‐analytic findings of Peng et al. (2016), which reported a correlation of *r* = 0.27 between working memory performance (primarily measured by span tasks) and algebra performance. Third, the observed correlations are comparable to those of other well‐established predictors of mathematical achievement that carry substantial theoretical or practical relevance. For instance, previous studies have reported similar or even smaller effect sizes for variables such as the home math environment (*r* = 0.13; Daucourt et al. 2021), approximate number acuity (*r* = 0.20; Chen and Li 2014), and math anxiety (*r* = −0.28; Barroso et al. 2021). Taken together, although the strength of the relationship between n‐back performance and algebraic ability falls within the small‐to‐moderate range, it may nonetheless carry meaningful theoretical and practical significance.

In addition, general intelligence, as measured by Raven's APM, was positively correlated with both n‐back performance (*r* = 0.23, *p* < 0.01) and algebraic performance (algebraic equation solving: *r* = 0.13, *p* = 0.06; algebraic word problem solving: *r* = 0.23, *p* < 0.01). Therefore, we examined whether n‐back performance provided incremental contributions to algebraic performance after controlling for general intelligence. To achieve this goal, we conducted two hierarchical regression analyses that included algebraic equation solving and word problem solving performance as dependent variables. The models included age, gender, and general intelligence in Step 1 and n‐back performance in Step 2. The analyses revealed that n‐back performance explained the additional variance in both algebraic equation solving (Step 1, *F*(3, 190) = 2.12, *R* ^2^ = 3.25%, *p* = 0.10; Step 2, *F*(4, 189) = 3.08, *R* ^2^ = 6.11%, *p* = 0.02; Δ*R* ^2^ = 2.87%) and algebraic word problem solving performance (Step 1, *F*(3, 190) = 6.97, *R* ^2^ = 9.92%, *p* < 0.001; Step 2, *F*(4, 189) = 7.97, *R* ^2^ = 14.43%, *p* < 0.0001; Δ*R* ^2^ = 4.52%) beyond the variance explained by age, gender, and general intelligence.

**FIGURE 2:**
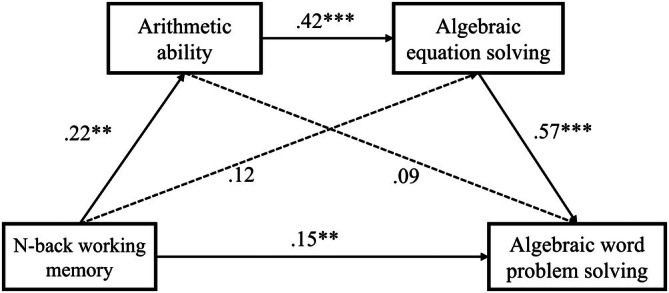
The sequential mediation model of n‐back performance as a predictor of algebraic word problem solving mediated by arithmetic ability and algebraic equation solving. Standardized regression coefficients are displayed for all paths. **p < 0.01; ***p < 0.001.

Next, we performed a mediation analysis to examine how n‐back memory influences algebraic ability (Figure 2). Because arithmetic ability constitutes the fundamental groundwork for solving algebraic problems (Tolar et al. 2009) and because solving algebraic word problems is dependent on the ability to solve algebraic equation problems, we built a comprehensive sequential mediation model with n‐back working memory as the predictor variable, algebraic word problem solving ability as the outcome variable, and arithmetic ability and algebraic equation solving ability as sequential mediator variables. The mediation analysis suggested that neither arithmetic ability (*β* = 0.02, bootstrapping CI = [−0.005, 0.06]) nor algebraic equation solving ability (*β* = 0.07, bootstrapping CI = [−0.005, 0.15]) alone mediated the relationship between n‐back working memory and algebraic word problem solving. However, the mediating pathway of n‐back working memory → arithmetic ability → algebraic equation solving ability → algebraic word problem solving ability was statistically significant (*β* = 0.05, bootstrapping CI = [0.02, 0.10]), supporting a serial mediation relationship. That is, greater n‐back working memory may positively contribute to arithmetic ability, which in turn promotes algebraic equation solving and ultimately results in better algebraic word problem solving performance. Furthermore, the analysis revealed that the direct influence of n‐back working memory on algebraic word problem solving was statistically significant (*β* = 0.15, bootstrapping CI = [0.05, 0.25]), which accounted for 51.4% of the variance in the overall effect of n‐back working memory on algebraic word problem solving (*β* = 0.28, bootstrapping CI = [0.15, 0.41]).

The findings of Study 1 can be summarized as follows. First, n‐back working memory positively contributed to algebraic abilities in solving equation problems as well as word problems, even beyond general intelligence. Second, the influence of n‐back working memory on algebraic word problem solving has two pathways, including a direct pathway and an indirect sequential mediation pathway (i.e., arithmetic ability and algebraic equation solving ability as sequential mediators).

## 3 Study 2

After confirming the association between working memory and algebraic ability, we conducted Study 2 to investigate whether training in working memory results in enhanced algebraic performance. Unlike Study 1's high school sample, Study 2 recruited college students to facilitate the intensive training schedule without disrupting classes. In addition, we purposefully employed a dual n‐back paradigm that presents two independent streams—a visuospatial stream (aligned with the spatial working memory measure used in Study 1) and an auditory‐verbal stream—because updating both streams simultaneously imposes a substantially heavier demand on the central executive than a single‐stream task. This dual‐modal load may more closely mirror the way algebraic manipulation requires learners to integrate continuous visuospatial tracking with concurrent inner verbalization.

### 3.1 Methods

#### 3.1.1 Participants and Procedures

The participants were 79 sophomore‐year college students who were recruited from Dali University. The mean age of the participants was 19.5 years (SD = 1.01), and 87.34% of the participants were girls. The Medical Ethics Committee of Dali University approved the study, and we obtained written informed consent from all participants.

All participants were randomly allocated to three groups: the experimental group (*n* = 28), active control group (*n* = 28), and passive control group (*n* = 23). The experimental group and the active control group participated in 20 days (4 days/week, 5 weeks) of n‐back working memory training. For the experimental group, the training was adaptive; that is, task difficulty (i.e., working memory load) increases with task performance. For the active control group, the memory load was constant across all training sessions. The participants in the passive control group did not receive any form of working memory training and participated only in regular school learning. One day before and 1 day after the training session, all participants completed two parallel forms of mathematical tests that were matched in length and difficulty. The participants in the experimental group, the active control group, and the passive control group received compensation of 100, 100, and 20 Chinese yuan, respectively, for their participation in the study.

The sample size of the study was predetermined with a prior power analysis in G*Power 3.1 (Faul et al. 2009). A total of 66 participants (i.e., 22 in each group) were needed to observe a medium‐sized interaction effect (*f* = 0.25; Cohen 1988) with an alpha level = 0.05 and power = 95%. We recruited more participants to counteract the impact of potential participant attrition.

#### 3.1.2 Tasks

##### 3.1.2.1 Training Task

The training for working memory involved a dual n‐back task, which was modified from Jaeggi et al.'s (2008) work. This training was conducted using the freely available online platform Brain Workshop 4.8.4 (brainworkshop.sourceforge.net).

**FIGURE 3:**
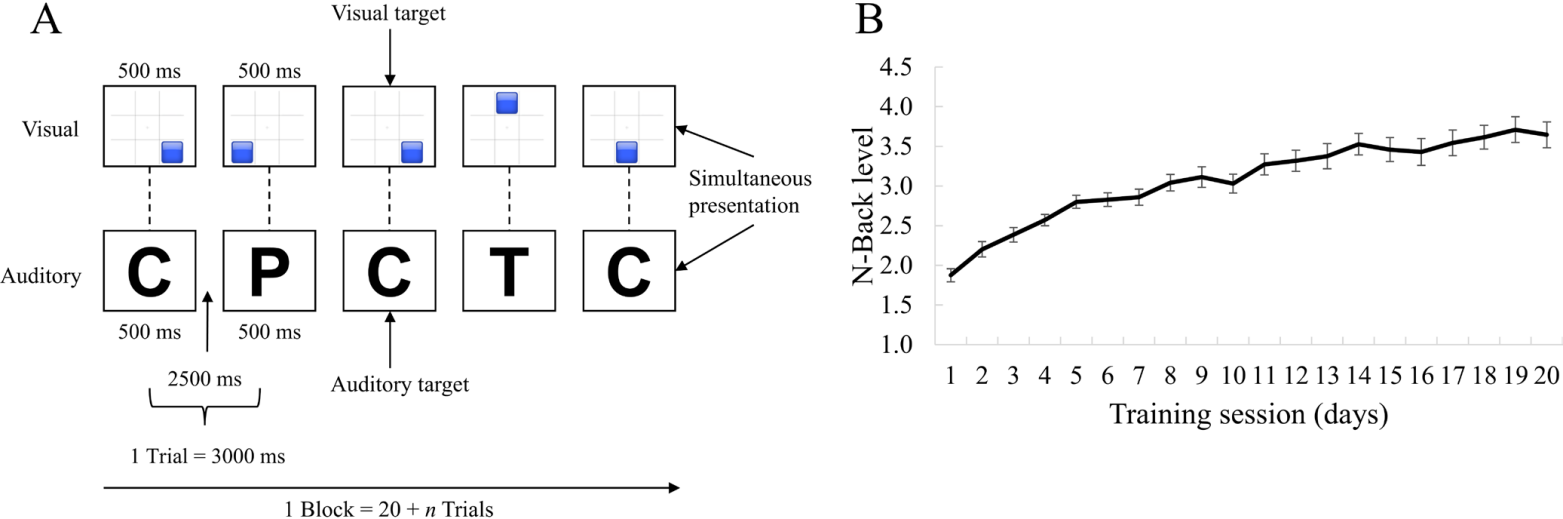
The procedure and performance of the adaptive n‐back task. (A) Training stimuli: Each trial presented a blue square in one of eight fixed screen locations alongside one of eight consonant sounds. Participants judged simultaneously whether the square's location and the consonant matched those presented *n* trials earlier, with *n* adapting between 1 and 5 based on individual performance. (B) Behavioral performance in the adaptive group—measured as the mean *n*‐level achieved—improved steadily over the course of training. Error bars represent one standard error above and below the mean.

This experiment employed both visuospatial and auditory stimuli (Figure 3A). The visuospatial stimuli comprised blue squares presented at one of eight positions evenly distributed around a constant white fixation cross at the center of a black screen. The auditory stimuli consisted of eight consonants (c, g, h, k, p, q, t, w) presented through auditory means. The participants were instructed to identify matches between the current stimulus and the stimulus presented *n* positions back in the sequence, with *n* varying based on the load level (1, 2, 3, 4, or 5). Each trial featured a 500 ms stimulus presentation followed by a 2500 ms interstimulus interval, after which the next stimulus was presented. Both visuospatial and verbal stimuli were presented simultaneously, so the participants were required to process each modality independently. Furthermore, the participants were required to respond to the visuospatial targets by pressing “A” and to the auditory targets by pressing “L.” No response was required in the absence of a target. The participants were instructed to make their judgments both quickly and accurately.

For the adaptive training group and active control groups, the participants were trained for 20 days for a total of 5 weeks; they could voluntarily choose any 4 days per week for training. The training each day included 20 blocks, and each block contained 20 + *n* (*n* = n‐back level) trials. The training was self‐paced, and the participants could determine their rest time between independent blocks. Generally, the duration of training for each participant per day was between 30 and 45 min.

For the adaptive training group, the level of difficulty of each training block was adapted by changing the level of n, which was also used to track the participants' performance (Jaeggi et al. 2008). The beginning n‐back level for each day was 2, and the subsequent n‐back level varied depending on the participants' performance based on Brain Workshop's algorithm (i.e., score = true positive/[true positive + false positive + false negative]): (1) increase the n‐back level: a score of 80% or greater in a block; (2) maintain the n‐back level: a score of 50% to 79% in a block; (3) decrease the n‐back level: three scores below 50% in three blocks (not necessarily in consecutive blocks). Each participant's daily behavioral performance was calculated as the average n‐back level across the 20 blocks they completed each day. For the active control group, the difficulty level of each training block remained constant, with the n‐back level consistently set to 1.

##### 3.1.2.2 Mathematical Tasks

We measured arithmetic ability in the pretest using the CFT. The algebraic equation solving and algebraic word problem solving tasks from Study 1 were used to measure algebraic ability with one unknown in the pretest.

To comprehensively capture the participants' algebraic ability, we additionally administered algebraic equation solving and algebraic word problem tasks with two unknowns. For the algebraic equation task with two unknowns, the participants were asked to solve systems of linear algebraic equations with two unknowns (e.g., “2X − Y = 5; X + Y = 16; X = ?; Y = ?”). This task included 60 questions on four A4 sheets (15 questions on each sheet). We also administered algebraic word problems with two unknowns, each of which could be represented as a pair of linear equations (e.g., “There are two kinds of ropes. The length of the first rope is twice the length of the second rope, and the second is 3 m shorter than the first. How many meters are each of these two ropes?”). This task included 24 questions on four A4 sheets (six questions on each sheet). The participants were given 6 min for the algebraic equation solving task and 10 min for the algebraic word problem solving task with two unknowns. For both tasks, the participants were instructed to solve the problems quickly and accurately. Before the formal test, the participants solved three sample practice problems from each subtest.

##### 3.1.2.3 Parallel Forms of Mathematical Tasks

To compare the changes in the participants' mathematical performance between the pretest and posttest, we developed parallel forms of all the math tests used in the pretest with the same length and comparable difficulty. The parallel forms were used in the posttest. To validate the comparability of the two forms of tests, we recruited an additional 28 college students (23 girls; mean age = 19.47, SD = 4.36) as participants. The participants completed the first set of parallel tests followed by a 5‐min break. They then proceeded to complete the second set of parallel tests. We found that the two sets of parallel tests demonstrated comparable difficulties and evidence of parallel form reliability (0.51 ≤ *r*s ≤ 0.81) (see Table S1 for details).

#### 3.1.3 Data Analysis

All statistical analyses were conducted using JASP (Love et al. 2019). Beyond traditional null hypothesis significance testing, we also used a Bayesian approach to detect the transfer effect of the training.

### 3.2 Results and Discussion

First, we replicated the positive associations between n‐back working memory performance and algebraic abilities observed in Study 1. That is, the n‐back working memory level on the first day of the adaptive group was positively correlated with all measures of algebraic abilities in the pretest, including performance in algebraic equation solving (one unknown: *r* = 0.42, *p* = 0.03; two unknowns: *r* = 0.42, *p* = 0.03) and algebraic word problem tasks (one unknown: *r* = 0.29, *p* = 0.14; two unknowns: *r* = 0.43, *p* = 0.02). Because the sample size was small (*n* = 28), we did not perform additional mediation analyses between n‐back working memory performance, arithmetic ability, and algebraic abilities, as in Study 1.

Next, as expected and in line with the extant literature (e.g., Jaeggi et al. 2008), we found that participants in the adaptive group demonstrated a training effect on working memory. Their average n‐back working memory level increased from an initial 1.87 on the first day to a final 3.65 on the final day, indicating a gain of 1.77 (Figure 3B). A paired‐samples *t* test comparing the initial and final levels also indicated a statistically significant and large training effect (*t*(27) = 14.23, *p* < 0.001, Cohen's *d* = 2.69), suggesting that our manipulation of working memory gain was successful.

We subsequently examined the most crucial research question, that is, whether the training effect of working memory would transfer to the mathematics domain. To address this issue, we performed five repeated‐measures ANOVA tests (see Table 2 for descriptive statistics of mathematical performances across different participant groups, along with *p* values and effect size measures from the ANOVA tests) with the five mathematical abilities as dependent variables and the Test (pretest/posttest) and Group (adaptive/active control/passive control) as independent variables. To detect the training effect, we focused on the Test*Group interaction effect and the corresponding post hoc comparisons (i.e., simple effects). In these comparisons, we conducted paired‐samples t‐tests to examine pretest–posttest differences within each group, and applied Bonferroni correction to control for the increased risk of Type I error due to multiple comparisons (Dunn 1961).

**TABLE 2:** Mathematical performances across different experimental conditions in Study 2.

Tasks		Adaptive group (*n* = 28)		Active control group (*n* = 28)		Passive control group (*n* = 23)		Repeated‐measures ANOVA: *p* value and partial *η* ^2^ (in parentheses)		
		Pretest	Posttest	Pretest	Posttest	Pretest	Posttest	Test	Group	Test × Group
Arithmetic ability		46.71 (13.28)	51.32 (13.25)	45.68 (11.26)	51.79 (12.18)	45.57 (11.69)	48.30 (12.45)	**< 0.001 (0.38)**	0.81 (0.01)	0.13 (0.05)
Algebraic equation solving	One unknown	22.36 (5.67)	21.57 (7.18)	21.14 (7.50)	21.11 (7.88)	21.78 (7.24)	22.30 (7.81)	0.84 (0.00)	0.86 (0.00)	0.58 (0.01)
	Two unknowns	27.96 (8.87)	32.43 (12.25)	28.75 (9.97)	30.04 (12.56)	28.39 (10.78)	34.87 (11.57)	**< 0.001 (0.27)**	0.75 (0.01)	**0.03 (0.09)**
Algebraic word problem solving	One unknown	10.25 (4.52)	9.93 (4.21)	9.46 (3.92)	8.86 (4.34)	10.09 (4.11)	9.52 (5.24)	0.10 (0.04)	0.70 (0.01)	0.91 (0.00)
	Two unknowns	12.75 (5.58)	17.00 (8.06)	11.82 (6.46)	16.32 (8.20)	12.78 (7.32)	17.04 (8.46)	**< 0.001 (0.41)**	0.88 (0.00)	0.98 (0.00)

*Note*: Values in pretest/posttest cells are presented as descriptive statistics (means and standard deviations); the *p* values in bold are statistically significant.

No statistically significant interaction effects were found for performance on the four mathematical tasks (i.e., CFT, algebraic equation solving with one unknown, algebraic word problem solving with one unknown, or algebraic word problem solving with two unknowns), indicating the lack of far‐transfer effects of training on these tests. In addition, although there was a statistically significant Test*Group interaction in the performance of algebraic equation solving with two unknowns, *F*(2, 76) = 3.87, *p* = 0.03, partial *η* ^2^ = 0.09, further examination of simple effects revealed that these interactions did not reflect transfer effects. That is, both the adaptive group (*t*(27) = 3.26, *p* = 0.003, Bonferroni‐adjusted *p* = 0.009, Cohen's *d* = 0.62) and the passive control group (*t*(22) = 5.45, *p* < 0.001, Bonferroni‐adjusted *p* < 0.003, Cohen's *d* = 1.14) showed significant performance improvements, with no discernible difference in improvement between them (*F*(1, 54) = 2.78, *p* = 0.10, partial *η* ^2^ = 0.05). The absence of performance improvement in the active control group (*t*(27) = 0.97, *p* = 0.34, Bonferroni‐adjusted *p* = 1.00, Cohen's *d* = 0.18) contributed to the observed interaction. Together, the repeated ANOVA tests did not support the existence of any transfer effects in the mathematical tasks.

Additionally, we performed Bayesian ANOVA (van den Bergh et al. 2020) to supplement the aforementioned ANOVA that showed null results of the training effects (i.e., the Test*Group interaction). This test directly assessed the likelihood of the alternative hypothesis (H1: the existence of a training effect) against the evidence for the null hypotheses (H0: the absence of a training effect). Specifically, the Bayes factors (BF_10_) were calculated to quantify the strength of evidence favoring H1 over H0. For instance, a BF_10_ of 0.1 suggests that the observed data are 10 times more likely under H0 than under H1. Following established guidelines for interpreting the magnitude of BF_10_ (Wagenmakers et al. 2018: 3–10, indicating moderate evidence for H1; 1–3, indicating anecdotal evidence for H1; 1/10–1/3, indicating moderate evidence for H0; 1/3–1 indicating anecdotal evidence for H0), the evidence generally favored the lack of Test*Group interaction effects on four mathematical tasks, including CFT (BF_10_ = 0.546), algebraic equation solving with one unknown (BF_10_ = 0.172), algebraic word problem solving with one unknown (BF_10_ = 0.119), and algebraic word problem solving with two unknowns (BF_10_ = 0.110). The Bayesian ANOVA approach also supported the conclusion that training effects were not transferred.

In summary, performance on the n‐back working memory task improved significantly, but this training effect did not transfer to the mathematical domain.

## 4 General Discussion

In the current investigation, we aimed to examine the link between n‐back working memory performance and algebraic ability in Chinese adolescents. Study 1 revealed a positive association between n‐back working memory performance and algebraic ability. Specifically, individuals with higher n‐back working memory scores demonstrated superior performance in solving both algebraic word problems and equations, and this relationship persisted after controlling for demographic variables and general intelligence. Furthermore, arithmetic ability and algebraic equation solving ability were found to sequentially mediate this relationship between working memory and algebraic word problem solving ability. Study 2 extended these findings by examining the effects of n‐back working memory training on algebraic performance. While the participants who underwent adaptive n‐back training showed significant improvements in working memory performance, these enhancements did not translate to better performance in algebraic tasks. Together, these studies demonstrated the positive relationship between n‐back working memory and algebraic ability while casting doubt on the effectiveness of working memory training for improving algebraic performance.

### 4.1 Association Between Working Memory Performance and Algebraic Ability

Despite a body of research that finds a positive correlation between working memory performance and algebraic ability (Cirino et al. 2022; Geary et al. 2015; Lee et al. 2004; Lee et al. 2009; Lee et al. 2011; Lee et al. 2018; Siegler et al. 2012; Tolar et al. 2009; Trezise and Reeve 2014), our study reveals new evidence and perspectives on this relationship.

First, our study contributes new insights to the existing body of research by exploring the link between n‐back working memory performance and algebraic ability beyond the traditional focus on complex span tasks to measure working memory performance. This contribution is particularly significant because it highlights the unique facets of working memory that the n‐back task captures (specifically, the task's emphasis on recognition processes and updating) in contrast to the processes predominantly engaged by complex span tasks (Redick and Lindsey 2013). Moreover, the emphasis of the n‐back task on the recognition and continuous updating of stimuli closely reflects the cognitive demands of algebra, which involves the persistent manipulation and updating of abstract symbols and operations. Together, our study provides new evidence of the pivotal role of working memory in solving algebraic problems (Peng et al. 2016). Future research may consider employing both complex span and n‐back tasks to achieve a more comprehensive understanding of the multifaceted working memory foundations of algebraic ability.

Second, based on the observed sequential mediation relationship, our study demonstrates how working memory affects performance in solving algebraic word problems via multiple pathways. One pathway is a direct influence: working memory can directly influence the ability to solve algebraic word problems. Another pathway is indirect: stronger working memory may improve arithmetic ability needed for algebra, which in turn benefits solving algebraic equations (see Tolar et al. 2009 for a similar finding using the complex span task). Furthermore, solving algebraic word problems also depends on the more fundamental ability to solve algebraic equations.

Based on these findings, we argue that schools and teachers could explore algebraic learning approaches tailored to individuals with different working memory capacities. The following strategies could be adopted. First of all, complex algebraic word problems should be broken into smaller, manageable segments (e.g., arithmetic problems, equation problems, the translation of word problems to equation problems), which allow students with varying working memory capacities to build foundational understanding before tackling more complex problems. Furthermore, adaptive technology should be integrated into practice sessions. Tailored problem sets that match a student's current understanding and working memory capability can encourage consistent progression and confidence. Finally, visual aids and interactive tools can be used to represent algebraic concepts and make abstract ideas more tangible. These resources can especially benefit students who have limited working memory capacity by enhancing their engagement and understanding.

### 4.2 The Far‐Transfer Effect of Working Memory Training

Although our investigation revealed a stable positive association between n‐back working memory and algebraic performance, we found that training in n‐back working memory did not produce a far‐transfer effect on algebraic performance. The absence of far‐transfer can be attributed to various possibilities, each of which suggests further directions for educational practice or future research.

The first and most plausible possibility is that working memory training inherently faces challenges in yielding far transfer. While early studies provided encouraging findings, recent meta‐analyses (Melby‐Lervåg and Hulme 2013; Melby‐Lervåg et al. 2016; Rodas et al. 2024; Sala et al. 2019; Sala and Gobet 2020) have cast doubts on the existence of far transfer. Our research in the algebraic domain reaffirms this perspective, demonstrating that even in the realm of highly complex algebraic cognition, heavily reliant on working memory, far transfer seems elusive. A more in‐depth explanation is that adaptive n‐back training enhances efficiency rather than capacity (von Bastian et al. 2022). Because capacity—the resource algebra relies on—remains unchanged, far transfer is unlikely. Consequently, if far transfer proves difficult to achieve, researchers and practitioners may not find it worthwhile to invest further effort in working memory training. Instead, resources appear better spent exploring algebraic learning approaches tailored to individuals with different working memory capacities.

The second possibility for the lack of observed far‐transfer effects to algebraic tests post‐training might be elucidated by Peng and Swanson's (2022) emphasis on the domain‐specific nature of working memory training. These authors argued that traditional, domain‐general working memory training does not guarantee far‐transfer effects to untrained tasks but rather demonstrates near‐transfer effects on tasks that share structural similarities. Hence, to boost adolescents' algebraic performance and ensure transfer, working memory training should embed algebra‐specific materials and strategies that align directly with core skills such as equation solving and symbolic manipulation. For instance, the intervention could use algebraic equations themselves as the stimuli in the training tasks. Future studies should determine whether algebra‐specific working memory interventions can measurably improve algebra performance, especially given the limited effects observed for traditional domain‐general training.

The third possibility is that individual differences might limit the potential for far‐transfer effects in working memory training on algebra. In our training study, the participants were college students who had passed the college entrance examinations, suggesting that they had higher working memory capacity and algebraic ability compared to those of the general population. Thus, they may have already reached the “functional threshold” required for solving algebraic problems, so further working memory gains would bring negligible benefits to algebraic performance. In contrast, individuals with limited working memory capacity or whose algebraic performance is still constrained by working memory bottlenecks might benefit more significantly from targeted training interventions (Zhang et al. 2018; Zhao et al. 2022). Investigating this possibility in future research could provide deeper insights into how cognitive training effects vary across developmental stages and baseline working memory profiles.

In summary, our investigations reliably identified a positive correlation between working memory performance and algebraic proficiency. Nevertheless, the expected enhancements in algebraic performance through working memory training were not observed, casting significant doubt on the widespread efficacy of such training in augmenting mathematical abilities. This inconsistency underlines the need for a thorough reevaluation of existing methodologies for working memory enhancement within the realm of mathematical education. To continue exploring the impact of working memory training on mathematical abilities, despite some recommendations to abandon this line of research altogether (e.g., Dougherty and Engle 2020), methodological reforms are essential (Lee 2023). Future research should focus on developing domain‐specific strategies (Peng and Swanson 2022) with adequate commitment and practice (Lee 2023) and designing personalized working memory training strategies that address learning needs in real educational contexts and the cognitive profiles of individuals. Moreover, a dedicated systematic review or meta‐analysis that examines potential moderators—such as training paradigm and participants' age—could shed light on the conditions under which these interventions succeed or fail, and thus help resolve the ongoing debate about their overall effectiveness.

## 5 Limitations and Future Directions

The current investigation has several limitations that deserve consideration and could be addressed in future studies.

First, our study's sample selection introduces certain limitations. For convenience in sampling, Study 1 and Study 2 recruited participants from two distinct developmental stages—middle adolescence (high school students) and late adolescence (university students), respectively. Although both groups share core adolescent characteristics, they exhibit important psychological differences (Meeus et al. 2010) that warrant testing our findings across a broader age range. Additionally, all participants were recruited from mainland China's educational system, which may differ in curriculum, instructional practices, and cultural context from those in other countries (Yang et al. 2025). This implies that future research should replicate these findings across samples from diverse national and cultural backgrounds to assess their generalizability (Lan et al. 2011).

Second, the study's assessment of algebraic ability was restricted to specific types of problems of elementary algebra and potentially overlooked other facets of algebraic thinking. Future investigations could incorporate a wider range of algebraic tasks, especially in intermediate and advanced algebra, which would enrich our understanding of the cognitive underpinnings of algebraic abilities.

Third, although we controlled for general intelligence when examining the relationship between working memory and mathematical abilities, other unmeasured third factors may still have played a role. For instance, because our algebraic tests were time‐limited and the n‐back task used a fixed stimulus pace, individual differences in processing speed could have influenced the observed correlation between working memory performance and algebraic ability (Trezise and Reeve 2014). Future studies should therefore incorporate processing speed measures to determine whether the correlation remains after partialling out speed‐related variance.

Fourth, because Study 1 used a cross‐sectional design, its serial mediation findings are only suggestive. To clarify how working memory, arithmetic ability, and algebraic ability influence one another over time, future research should adopt longitudinal designs.

Fifth, although Study 2 did not reveal any far‐transfer effects of working memory training on algebraic ability, there is still considerable room to refine training protocols and to improve the evaluation of their efficacy before we close the book on the enterprise (Lee 2023). As we noted earlier, one promising avenue is to explore domain‐specific working memory training using algebra‐related materials (Peng and Swanson 2022). A second line of inquiry concerns the long‐term impact of such training, particularly in light of one study indicating that gains in mathematical performance may emerge only after a delay rather than immediately (Holmes et al. 2009). A further direction would be to employ cognitive neuroscience techniques to examine how changes in the brain caused by working memory training (Thompson et al. 2016) are linked to the networks that support algebraic thinking, because neural indices can sometimes be more sensitive to training‐induced changes—and may emerge prior to observable behavioral gains (Brooks et al. 2020).

## 6 Conclusion

Adolescents' ability in algebra plays a crucial role in both their academic achievements and their future career prospects. Our investigation revealed the critical influence of working memory on algebraic ability, indicated by a significant positive correlation between these two factors. However, our results question the direct benefits of working memory training programs for algebraic enhancement, as an increase in working memory performance did not translate into improvements in algebra. Consequently, until more effective ways of harnessing working memory training to boost algebraic ability are identified, educators may wish to devote more effort to fostering algebraic proficiency by creating optimal learning environments and offering support that is carefully tailored to meet students' individual working memory needs. At the same time, researchers should remain open to testing the effects of rigorously designed, math‐specific, and personalized working memory interventions on algebraic ability.

## Ethics Statement

The Medical Ethics Committee of Dali University approved the study. Written consent was obtained from high school students and their parents in Study 1, and from university students in Study 2.

## Conflicts of Interest

The authors declare no conflicts of interest.

## Supporting information


**Table S1:** Evidence for parallel reliability of mathematical tests in Study 2.

## Data Availability

The data that support the findings of this study are available from the corresponding author upon reasonable request.
