# Supplementary material for: Unraveling the Link Between n‐Back Working Memory and Algebraic Ability in Adolescents: Correlations and Training Effects
Source: Psych J. 2025 Aug 7;14(6):926–39. doi: 10.1002/pchj.70047 (PMC12702589; doi:10.1002/pchj.70047)
Supplement: Supplementary file 1 — Table S1: Evidence for parallel reliability of mathematical tests in Study 2. [file PCHJ-14-926-s001.docx]

Supplementary materials

Table S1. Evidence for parallel reliability of mathematical tests in Study 2

| Tasks | |  | Form A | |  | Form B | |  | Pearson correlations  between two parallel forms |
| --- | --- | --- | --- | --- | --- | --- | --- | --- | --- |
|  |  |  | Mean | SD |  | Mean | SD |  |  |
| Arithmetic ability | |  | 53.96 | 9.04 |  | 53.00 | 9.04 |  | .63*** |
| Algebraic  equation solving | one unknown |  | 26.71 | 7.40 |  | 25.11 | 7.65 |  | .81*** |
|  | two unknowns |  | 31.96 | 12.20 |  | 38.11 | 12.33 |  | .51** |
| Algebraic  word problem solving | one unknown |  | 10.29 | 3.68 |  | 10.68 | 5.05 |  | .66*** |
|  | two unknowns |  | 16.89 | 7.71 |  | 16.71 | 9.44 |  | .71*** |

Note: ** *p* < .01; *** *p* < .001
